# Nanostructured Lipid Carriers: A potential drug carrier for cancer chemotherapy

**DOI:** 10.1186/1476-511X-11-159

**Published:** 2012-11-20

**Authors:** Subramanian Selvamuthukumar, Ramaiyan Velmurugan

**Affiliations:** 1 Department of Pharmacy, Annamalai University, Annamalai nagar, 608002, Tamilnadu, India

**Keywords:** Nanostructured lipid carriers, cancer chemotherapy, controlled drug release, increased drug load, stability, tissue target

## Abstract

Nanotechnology having developed exponentially, the aim has been on therapeutic undertaking, particularly for cancerous disease chemotherapy. Nanostructured lipid carriers have attracted expanding scientific and commercial vigilance in the last couple of years as alternate carriers for the pharmaceutical consignment, particularly anticancer pharmaceuticals. Shortcomings often came across with anticancer mixtures, such as poor solubility, normal tissue toxicity, poor specificity and steadiness, as well as the high incidence rate of pharmaceutical resistance and the rapid degradation, need of large-scale output procedures, a fast release of the pharmaceutical from its carrier scheme, steadiness troubles, the residues of the organic solvents utilized in the output method and the toxicity from the polymer with esteem to the carrier scheme are anticipated to be overcome through use of the Nanostructured Lipid Carrier. In this review the benefits, types, drug release modulations, steadiness and output techniques of NLCs are discussed. In supplement, the function of NLC in cancer chemotherapy is presented and hotspots in research are emphasized. It is foreseen that, in the beside future, nanostructured lipid carriers will be further advanced to consign cytotoxic anticancer compounds in a more efficient, exact and protected manner.

## Introduction

In the pharmaceutical breakthrough today, the new technologies lead to find numerous new mighty compounds. To double-check progress in drug therapy the development of new drugs solely is not sufficient. Poor water solubility and insufficient bioavailability of the new drug substances are very widespread issues encountered. Thus, there is an expanding need to develop a pharmaceutical carrier scheme that overcomes these matters. This carrier scheme should be free of toxicity, have an adequate pharmaceutical loading capability and the possibility of pharmaceutical targeting and controlled release characteristics. The system should provide chemical and personal steadiness for the incorporated pharmaceutical. The feasibility of the production technique and as well the affordability should also be accessible [1-3].

Colloidal systems enquired have their own disadvantages. Shortcomings often encountered with the colloidal schemes such as liposomes, micro and nanoemulsions, nanocapsules, nanosponges and polymeric nanoparticles are the rapid degradation by the pH of the stomach or by the intestinal enzymes and the bile salts if taken orally, restricted physical and chemical steadiness throughout storage [4-6], need of large-scale output methods, a fast release of the drug from its carrier system, stability difficulties, the residues of the organic solvents used in the output method, the toxicity from the polymer [7,8] and numerous to say. All of these points make these colloidal carriers not optimal as a pharmaceutical carrier system.

SLN have been presented as an alternate carrier scheme to emulsions, liposomes and polymeric nanoparticles. SLN are formulated from solid lipids only. Therefore, after groundwork at smallest a part of the particles crystallizes in a higher energy modification (α or β`). Throughout storage, these modifications can transform to the low power, more organised β modification. Due to this modification high degree of alignment, the number of imperfections in the crystal lattice is small; this directs to drug expulsion [9].

NLC have been developed to overwhelm the drawbacks affiliated with SLN. They are advised to be the second lifetime of lipid nanoparticles. Contrasted to SLN, NLC show a higher loading capability for hardworking compounds by conceiving a less organized solid lipid matrix, i.e. by blending a fluid lipid with the solid lipid, a higher element drug stacking can be achieved. Thus, the NLC have an expanded drug stacking capacity in evaluation to SLN and the likelihood of drug expulsion during storage is less [2,10-12]. NLC have also a lower water content of the element suspension and a less inclination of unpredictable gelation [12-14].

NLC disclosed some benefits contrasted to the other colloidal carrier schemes. They supply a controlled pharmaceutical issue and an increase in chemical stability of the incorporated drugs. Furthermore, they are protected carriers which can be produced effortlessly on large scale [2,12,15-17].

### Nanostructured lipid carrier’s classification

It is well renowned from the study of suppositories that highly organized crystalline lipid matrices will lead to pharmaceutical expulsion. Lipid nanoparticles and microparticles made from blends of solid lipids can experience this, especially when nanoparticles are arranged from highly purified lipids, for example, tristearin [18].

**Figure 1:**
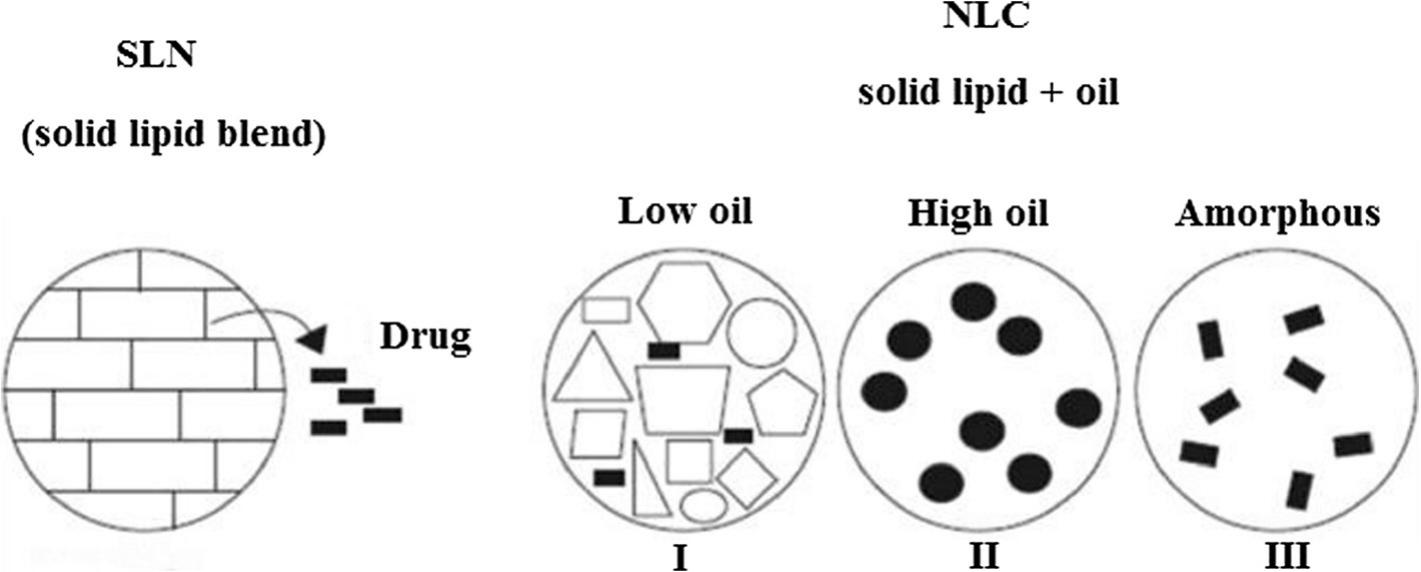
**SLN with high crystallinity and Different types of NLC.** I – Highly imperfect matrix, II – Multiple O/F/W type, III – non-crystalline amorphous NLC.

The formation of highly ordered βi or β modifications, particularly during storage, departs little space for pharmaceutical molecules, and the expulsion of pharmaceuticals leads to drug crystals in suspensions and solid dosage forms. To avoid this difficulty, the particles should have a controlled nanostructure that boasts enough space to accommodate the pharmaceutical. Four distinct approaches were taken for an optimized nanostructure of NLCs. In kind I, solid lipids and fluid lipids (oils) are blended. The difference in the organizations of the lipids and exceptional requirements in the crystallization process lead to a highly disordered, imperfect lipid matrix structure proposing space for drug substances and amorphous clusters of pharmaceuticals (Figure 1, I).

In general, drug solubility is higher in fluid lipids than in solid lipids. Founded on this, particles were produced with a high content of liquid lipids (oils). Throughout the production method, the liquid lipid particles (nanoemulsions) are chilled from the molten state to room warmth to crystallize and pattern solid particles. At high oil concentrations a miscibility gap of the two lipids (solid lipid plus oil) happens throughout the chilling phase, leading to stage separation, that means precipitation of minute oily nanocompartments (Figure 1, II). In this multiple oil/fat/water, kind II drug can be accommodated in the solid, but at increased solubility in the oily components of the lipid matrix.

In type III, lipids are blended in a way that stops them from crystallizing. The lipid matrix is solid, but in an amorphous state (Figure 1, III) [19]. The nonattendance of crystallization avoids pharmaceutical expulsion by crystallization. Lipid particles are preferentially matched to incorporate lipophilic pharmaceuticals; hydrophilic drugs can only be incorporated at a low percentage (however, this is still sufficient for highly powerful peptides and proteins). In a further variation of the lipid matrix, water-soluble pharmaceuticals were conjugated with a lipid, thus forming a water-insoluble lipidic conjugate. The lipid conjugate dust was dissolved and processed in the same way as the other types to yield a lipid drug conjugate (LDC) nanoparticle [20] counting on the conjugate, this lipidic conjugate has a pharmaceutical stacking of up to 30–50% for water-soluble pharmaceuticals. Conjugation is presented by salt formation or covalent linkage.

### Modulation of Pharmaceutical Release

**Figure 2:**
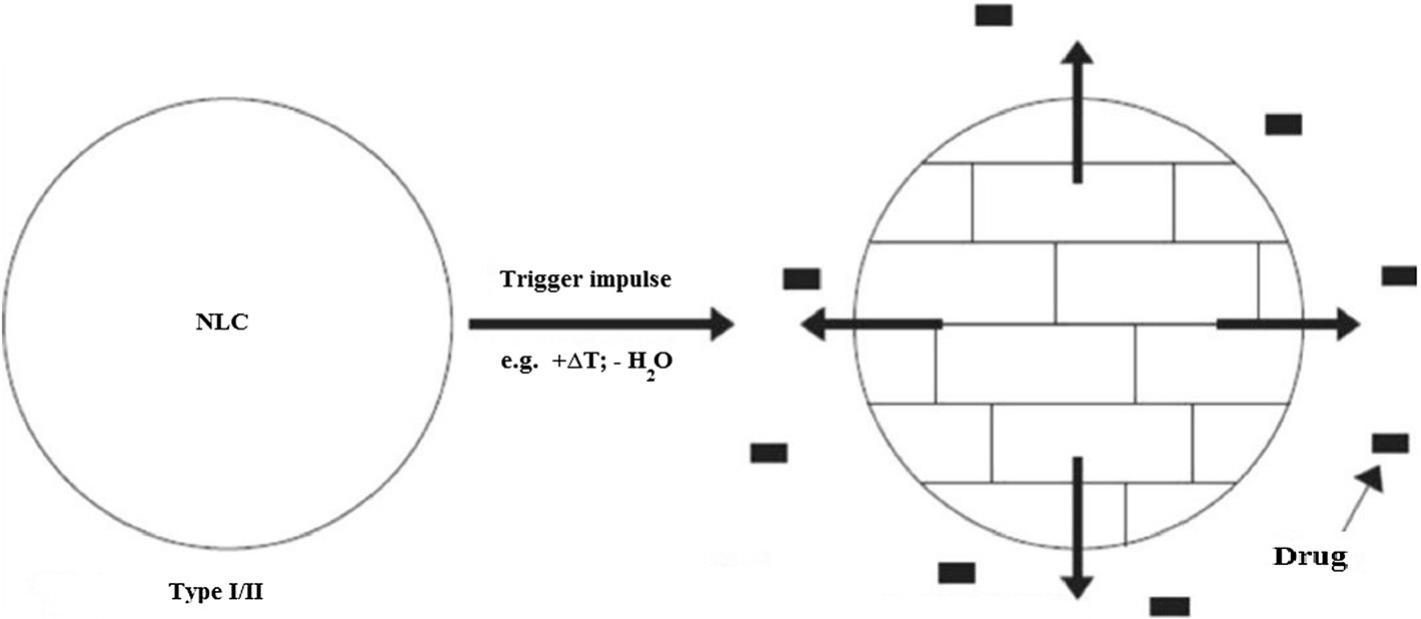
Triggered pharmaceutical releases from NLC by starting the alteration from a highly disorganized lipid structure to more organized stable modifications.

Pharmaceutical issue from lipid particles happens by diffusion and simultaneously by lipid element degradation in the body. In some cases it might be desirable to have a controlled very quick release going after diffusion and degradation. Perfectly this issue should be triggered by an impulse when the particles are administered. NLCs accommodate the pharmaceutical because of their highly unordered lipid organizations. By applying the initiate impulse to the matrix to alter in a more ordered structure, such a desired blew pharmaceutical release can be started. NLCs of certain organizations can be triggered this way; for demonstration, when applying the particles to the skin incorporated in a cream. Boost in warmth and water evaporation leads to a boost in pharmaceutical issue rate (Figure 2). Founded on these cyclosporine-lipid particles are under development to heal psoriasis [21,22]. The cream itself is saturated with cyclosporine, as well as a cyclosporine-loaded NLC contained in the cream. After application to the skin, accelerated issue from the lipid particles should lead to a supersaturated scheme (similar to micro emulsions, but without high surfactant engrossment) premier to an improved penetration of cyclosporine into the skin.

### Long-Term Stability

**Figure 3:**
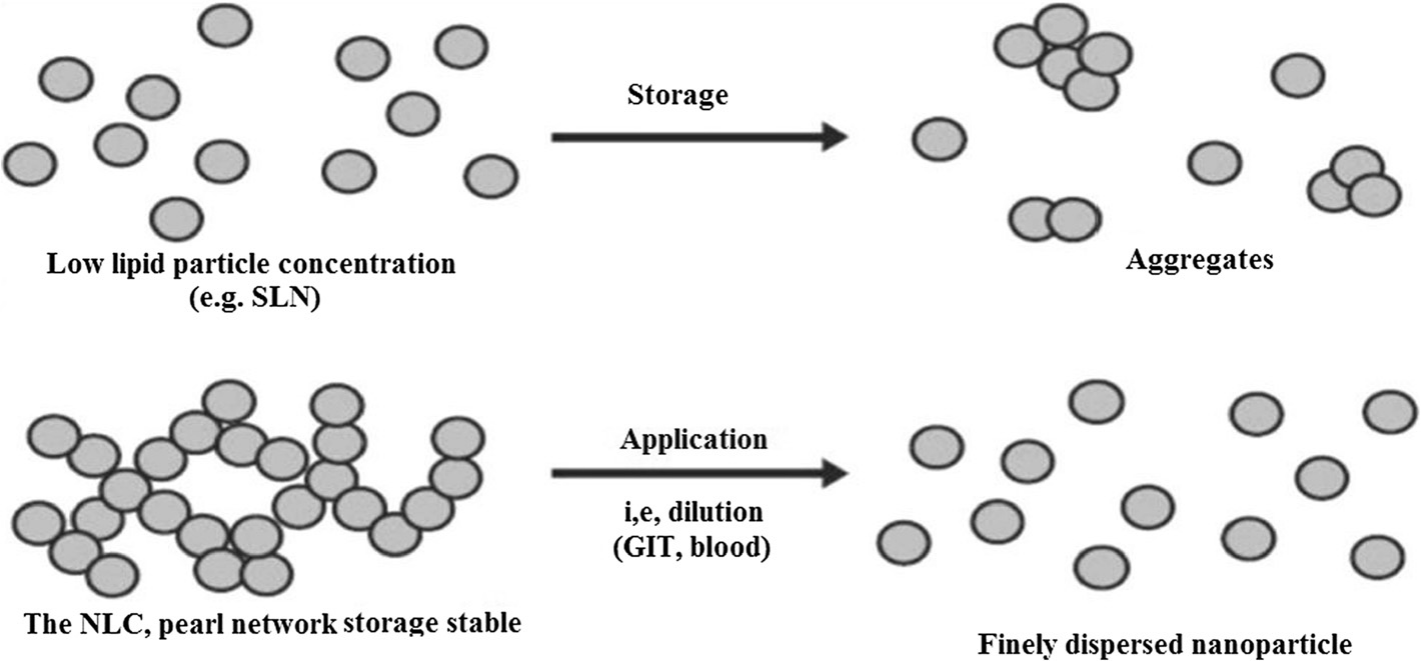
Aggregation method in reduced intensified dispersions (upper) and pearl-like network in NLC dispersions with stabilizing effect.

**Figure 4:**
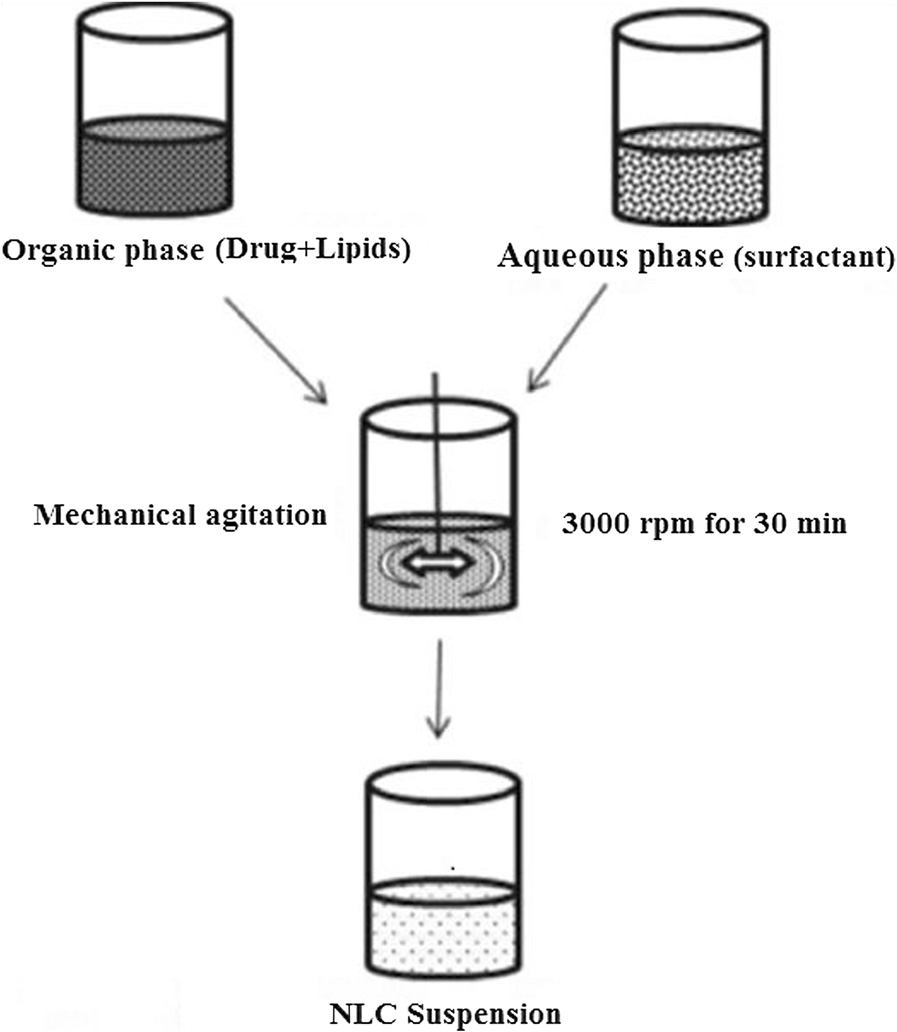
Output of NLC Nanosuspension.

During long-term storage of dispersions, element aggregation can happen. Aggregation and case formation were described for SLNs [23]. Lone particles diffuse in the dispersion medium; collision of particles can lead to perikinetic flocculation (Figure 3a). In the highly intensified NLC dispersions the particles pattern a ‘pearl-like network’, thus the particles are in a repaired place and will not undergo collision and perikinetic flocculation. After management of the particles and dilution with fluids (gastrointestinal fluids, for example), the mesh is decimated issuing single, non-aggregated particles (Figure 3b). Lipid particle dispersions were produced at identical surfactant concentration, but with reduced lipid content (be reduced 30%, outside patent coverage) and with 35% lipid. The low element dispersion aggregated during storage time, the gel-like NLC dispersion remained stable throughout storage and, after dilution, and single particles were obtained displaying no size boost [24] (Figure 4).

### Output of NLCs

NLCs can be made by various traditional dispersion methods. The favored output method is solvent diffusion method which was even said to be the most befitting procedure for the preparation of the nanoparticles [25]. Few other methods are Homogenization technique, double emulsion, supercritical fluid technology etc. High pressure homogenization technique is highlighted here. First the lipid phase is melted. Then the drug is dissolved in the molten lipid preparing a drug containing lipid melt. This melt is then dispersed in an aqueous surfactant solution heated at the same temperature, using a high speed stirring. The obtained pre-emulsion is then homogenized. The hot o/w emulsion obtained is solidified by cooling down to room temperature. The solidification of the Nano emulsions produced Nanostructured lipid carriers. The highly concentrated NLC dispersions are highly viscous, gel-like or pasty. These systems have no flowability. To make an 80% NLC dispersion, a multistep production method is directed. First, a 50% SLN dispersion is made by high-pressure homogenization. One hundred grams of such dispersion comprises 50 g of lipid and 50 g of water. In the next step, 10 g of lipid is supplemented, which is dispersed by high-speed stirring in the remaining 50 g of water. This outcome in 110 g of dispersion encompassing 60 g of lipid (55%) and a fresh 50 g of water. In the next step, another 10 g of lipid is dispersed in this 50 g of water stage and so on until a lipid content of 80% is come to. Large-scale production of NLC is easily possible. High-pressure homogenizers are accessible to method one ton and more per hour. There are furthermore no regulatory obstacles because these appliances are acknowledged in output lines for parenteral.

### Drawbacks encountered in cancer chemotherapy and the use of NLC

Numerous forms of malignancy, especially solid tumors, have historic presented important challenges to accepted chemotherapy. Regardless of the advancement in therapeutic cocktails, the conclusion of chemotherapy remains unsatisfactory. For demonstration, the response rate of pancreatic cancerous disease, oesophageal cancerous disease and ovarian cancerous disease to chemotherapy are well underneath 20% [26]. Even in patients with malignancies that are more perceptive to chemotherapeutic agencies, e.g. Breast cancerous disease, the clinical conclusions are generally underneath expectation [27]. In evaluation to other pharmaceutical classes, cytotoxic anticancer pharmaceuticals present unique difficulties such as poor specificity, high toxicity and susceptibility to induce pharmaceutical resistance. Conventionally administered cytotoxic agencies often extensively and indiscriminately join to body tissues and serum protein in a highly unpredictable kind. Only a little part of the drugs comes to the tumor location [28]. This may both reduce the therapeutic efficacy and boost systemic pharmaceutical toxicity. Furthermore, even though cytotoxic drugs perfectly should only murder cancerous disease units, in reality they are also toxic to non-cancerous units, particularly to rapidly splitting up units, e.g. skeletal part marrow units and units of the gastrointestinal tract. The normal tissue toxicities regularly occur even when benchmark therapeutic doses of anticancer drugs are administered. The poor specificity of cytotoxic drugs in terms of both pharmaceutical biodistribution as well as pharmacology at the cellular level impersonates a significant challenge to productive anticancer remedy. This poor side effect profile of cytotoxic pharmaceuticals has considerably diminished the therapeutic worth of the pharmaceuticals. Counting on the alternative of drugs, different organs or tissues can be annoyed or impaired by the non-specific activity of the cytotoxic agencies [29,30]. While edge effects such as nausea, vomiting, fatigue, and hair loss are commonly caused by almost all cytotoxic pharmaceuticals, some side-effects are drug-specific. For demonstration, anthracyclines can origin cardiotoxicity [31,32]. Some of these side-effects are cumulative and life-threatening. Cytotoxic drugs normally display a steep dose-response bend and high dose intensity is needed to double-check therapeutic success [33]. This leads to a tough dilemma for clinicians to select between high drug doses with a high risk of usual tissue toxicities or reduced pharmaceutical doses with reduced probabilities of therapeutic answer. In addition, there are other obstacles to the optimal presentation of conventional chemotherapy. Cancerous disease units workout a kind of mechanisms at cellular grade to weaken the toxicity of chemoherapeutic agencies they are revealed to. These protecting against mechanisms are sometimes categorized as “cellular” pharmaceutical resistance. The most prominent one is the multidrug resistance (MDR) phenotype, which engages hardworking efflux of a broad variety of cytotoxic pharmaceutical substances out of the cytoplasm by membrane- bound transporters [34-36]. In supplement to cellular mechanisms, cancerous disease units in solid tumors are inclined to be more resistant to chemotherapy than non-aggregating cancerous disease cells due to diverse pharmaceutical permeation obstacles, which makes it tough to achieve high intratumoral pharmaceutical concentration in solid tumors [34]. This type of drug resistance, or occasionally mentioned as “non-cellular” drug resistance, may farther lead to compromised clinical outcomes even though an anticancer drug has powerful in vitro efficacy. We believe NLC offer a pledge to overcome at least some of these obstacles.

### Rationale of utilizing NLC for anticancer pharmaceutical delivery

As the clinical outcomes of cancerous disease are usually underneath the expectations, Nanostructured lipid carriers could be used as an alternative drug carrier in the anticancer drug delivery.

In evaluation to other drug classes, a tumor is often affiliated with a defective, leaky vascular architecture as a result of the badly regulated environment of tumor angiogenesis. In addition, the interstitial fluid inside a tumor is generally insufficiently drained by a badly formed lymphatic system. As a outcome submicron-sized particulate issue may preferentially extravasate into the tumor and be kept there. This is often mentioned as the “enhanced permeability and keeping” (EPR) effect [37]. This EPR effect can be taken benefit of by a correctly conceived nanoparticle scheme such as NLC to achieve passive tumor targeting. By doing so, the aforementioned poor tissue specificity difficulty can be partially solved. Furthermore, with the improvement in surface-engineering expertise, the bio distribution of NLC can be further manipulated by modifying the exterior physico-chemical properties of NLC to goal them to the tissue of interest [38]. This maximizes the allowance of pharmaceutical that can come to the aimed at tumor sites and minimizes systemic pharmaceutical toxicity.

Like other types of drug carrier used for cytotoxic drug consignment, such as polymeric systems and liposomes, NLC have the benefits of personal stability, defense of labile pharmaceuticals from degradation, controlled issue, and easy preparation. At the identical time, NLC avoid some matters came across by those pharmaceutical consignment systems. NLC do not carry the somewhat high cost required for large scale production of liposomal formulations. They have fewer storage and drug leakage difficulties compared to schemes such as liposomes. The significant toxicity and acidity affiliated with a number of biodegradable polymeric materials are also not discerned in NLC. Cytotoxic anticancer pharmaceuticals are known for their heterogeneity. They constitute a class of compounds with highly varied molecular structure and physicochemical properties [26]. A carrier material, such as polymers, that can join one cytotoxic drug may not be compatible with another one. The versatility of NLC is thus exceedingly precious for the encapsulation of cytotoxic mixtures. In addition to the natural proficiency of NLC to effectively incorporate lipophilic mixtures, the more lately evolved variations of NLC (e.g. polymer-lipid hybrid nanoparticles, lipid-drug conjugate nanoparticles, to be considered subsequent) farther elaborate the function of NLC for the encapsulation of hydrophilic, ionic mixtures. numerous biocompatible and biodegradable lipids (e.g. triglycerides such as tristearin, tripalmitin, trilaurin; hard fats such as Witepsol sequence, glyceryl behenate, cetylpalmitate, lipid acids such as stearic acid, palmitic unpleasant) are accessible for groundwork of NLC [39]. In supplement, NLC formulations are compatible with most emulsifiers (e.g. poloxamer 188, polysorbate 80, lecithin, sodium glycocholate) accepted by pharmaceutical regulatory bureaus [7,39]. This versatility of NLC as a pharmaceutical carrier makes NLC a potential generic stage for the consignment of diverse anticancer cytotoxic agents.

### Function of NLC for cytotoxic pharmaceutical consignment- advancement and strategies

Number of cytotoxic agencies (verapamil, cyclosporin-A and N {4 [2 (1,2,3,4tetrahydro6,7dimethoxy-2-isoquinolinyl)-ethyl]phenyl}9,10dihydro5methoxy 9-oxo-4-acridine carboxamide (GG918)) which own undertakings to overcome the multidrug resistance phenotype in units resistant to cytotoxic drugs have been laden into these schemes. A number of strategies have been evolved to meet the major trials in anticancer pharmaceutical consignment encompassing i) encapsulation of water-soluble anticancer compounds, ii) advanced command of the rate and span of drug release and iii) avoidance of systemic clearance of NLC by the reticuloendothelial scheme (RES).

### Encapsulation of water-soluble anticancer compounds

Despite of the alternative of NLC groundwork procedure, the lipid needs to be dissolve and dispersed into lipid droplets of submicron dimensions in aqueous medium, whether by mechanical or thermodynamic means, to permit formation of nanoparticles. The drug to be encapsulated desires to amply partition into the dissolved lipid droplets to achieve good pharmaceutical stacking [38]. It is thus conceivable that lipophilic anticancer mixtures can be effectively incorporated into NLC because they are anticipated to partition well in lipids. In addition, some lipophilic pharmaceuticals are more routinely utilized in salt types because these salts can be conveniently weak and administered utilizing routinely accessible water-based vehicles (e.g. 0.9% saline) [40]. On the other hand, there are also a number of hydrophilic, water dissolvable agents which are to be incorporated. An approach to allow significant incorporation of these water-soluble anticancer pharmaceuticals into NLC is apparently vital. Several schemes have been devised to encapsulate ionic salts of cytotoxic pharmaceuticals. One engages the supplement of organic contradict ions to pattern ionic in twos with the charged drug substances described by Gasco and coworkers [41]. The group utilized decyl phosphate or hexadecyl to enhance loading of the saline types of doxorubicin and idarubicin into SLN made of stearic unpleasant. Important improvements in the partitioning of the drugs into the lipid were illustrated. For certain highly water-soluble cytotoxic agencies, that do not bear any ascribe, all of the aforementioned procedures, which are based on ascribe neutralization, may not be applicable. One likely answer is to arrange lipophilic drug derivatives and incorporated it in NLC.

### Advanced command of the rate and span of drug release

Lipid based carrier schemes evolved in early 90's often release pharmaceuticals in a non-uniform, biphasic latest trend. Fast issue of a large dose of drug is usually discerned primarily, pursued by slow and incomplete release of drug [42]. The quick initial pharmaceutical issue, upon submission, is often recounted as a “burst effect” or “burst release”. It presents a potentially significant difficulty in anticancer drug consignment because of the powerful potency of cytotoxic compounds. A large dose of anticancer drug quickly unpacked into the systemic circulation (if administered systemically) or released beside the localized injection site (if administered loco- regionally) can possibly origin an important wellbeing hazard. In NLC, crystallinity of the lipid is decreased by mixing spatially incompatible solid lipids or solid lipids with small amounts of oils. The proficiency of NLC to have a decreased blew effect and an increased payload of certain oil-soluble pharmaceuticals makes them a fairly attractive vehicle for anticancer drug consignment. Drug issue can be slow and incomplete from SLN after the initial burst. For instance, the release was less than 0.1% in different schemes consigning anticancer pharmaceuticals encompassing doxorubicin, idarubicin and paclitaxel [41,43].

Expanded drug issue is generally considered to be an affirmative characteristic in most types of treatment. Although, in periods of cancerous disease chemotherapy, its influence is difficult to forecast. It has been shown that relentless exposure to sub-optimal levels of cytotoxic agencies may induce the sign of membrane-associated pharmaceutical transporters (e.g. P-glycoprotein (P-gp)) [44,45] and therefore renders cancerous disease units more drug-resistant. A NLC scheme that may release anticancer pharmaceutical much quicker without a strong blew release may bypass this theoretical risk.

### Development of NLC schemes able to avoid clearance by reticuloendothelial system

Following intravenous management, pharmaceutical delivery schemes such as polymeric nanoparticles and liposomes are rapidly unblocked from the systemic circulation by the RES, furthermore renowned as mononuclear phagocyte system. RES is a part of the immune scheme consisting of phagocytic cells, which generally reside in the spleen, lymph nodes, and also in the liver in the pattern of Kupffer units. These phagocytic units can eliminate pharmaceutical carriers identified as foreign objects inside minutes [44,45]. RES clearance of pharmaceutical carriers would be useful only if lymph nodes, liver or spleen were the goal tumor site. Although for other cancers, RES clearance is expected a foremost barricade to systemic cytotoxic pharmaceutical consignment by NLC. When a particulate pharmaceutical carrier is encased with hydrophilic polymers the carrier becomes more resistant to RES clearance. This is partly because these polymers favor exterior adsorption of proteins that suppress opsonisation in vivo [46]. This type of polymer-coated drug consignment scheme is often mentioned to as “stealth”, for their proficiency to evidently evade the surveillance of immune scheme, or more rightly “long – circulating” drug carriers. This kind of scheme is able to stay in the circulation for prolonged times, with a half-life of a couple of hours in rodent forms to as high as 55 h in human subjects [46-48]. Long-circulating NLC formulations were prepared by coating the nanoparticles with a polymer. To facilitate more protected attachment of the hydrophilic polymer up on the surfaces of the lipid cores, polymer molecules were pre-conjugated to lipophilic moieties to form amphiphilic stealth outer layer agents. By increasing the engrossment of the outer layer agency it was shown that the zeta potentials of nanoparticles were decreased and their mean diameters were increased [49]. The presence of hydrophilic polymer substances adsorbed up on the particle exterior using the conjugated lipophilic moieties conspicuously shielded the exterior ascribes and increased the hydrodynamic volumes of the nanoparticles. The use of higher concentration of lipid and polymer furthermore moderately expanded the polydispersity catalogue. Hence, one should accept in mind that outer layer NLC with a stealth agency may affect more than just the clearance rates by the RES. Diverse physicochemical properties of the element systems are furthermore modified, which may considerably sway NLC presentation, security or formulation stability.

## Conclusion

Nano particulate pharmaceutical delivery systems offer a large influence to overwhelm some of the obstacles to effectively target a number of varied cell types. Despite the recent advancement in the therapeutic, significant challenges still present in the area of cell cancerous disease. Routinely utilized chemotherapy have given unsatisfactory outcomes, as the therapy is deleterious to patient wellbeing by making patients more susceptible to other infections and often cause death by weakening the immune system of the persevering body. Thus, a budding interest in nanotechnology has been developed remarkable number of advancements in latest years with a main aim on current cancer therapy.

Nanoparticulate delivery of anticancer pharmaceuticals to tumor tissues can be accomplished by organizing the nanodrug carrier. Nanotechnology is anticipated to play a progressively important function in the diagnostics, prognostics and administration of aimed at cancerous disease treatments. Nanotechnology is very quick expanding area of study foreseen to lead to development of innovative, sophisticated applications which recognize skin cancer units, consign drugs to target tissue, describing conclusion of treatment, and supervise intracellular changes to help avert precancerous cells from evolving malignant. The future remains exciting and wide open for on-going efforts by scientists, investigators and medical staff can genuinely ensure to do big things using the very little. The perfect consignment scheme would be targeted and accurately controlled. To goal nanoparticles to the desired tissues, a number of procedures have been evolved. These include physical means such as controlling the dimensions, ascribe and hydrophobicity of the particles. In supplement, targeting substances, such as antibodies and peptides, that identify exact cell exterior proteins and receptors, can be conjugated to the nanoparticle exterior to expressly goal specific cell types.

## Competing interests

The authors declare that they have no competing interests.

## Authors’ contributions

SS was responsible for the layout and initial idea for the manuscript and participated in researching the literature and critical discussion of the work. RV wrote the manuscript. Both the authors read and approved the final manuscript.

## References

[B1] Barratt GM Therapeutic applications of colloidal drug carriers Pharm Sci Technolo Today 2000 3 163 171 10.1016/S1461-5347(00)00255-8 10785658

[B2] Mehnert W Mader K Solid lipid nanoparticles: production, characterization and applications Adv Drug Deliv Rev 2001 47 165 196 10.1016/S0169-409X(01)00105-3 11311991

[B3] Mainardes RM Silva LP Drug delivery systems: past, present, and future Curr Drug Targets 2004 5 449 455 10.2174/1389450043345407 15216911

[B4] Gregoriadis G Florence AT Patel HM Florence AT, Chur GG Liposomes in drug delivery Drug Targeting and Delivery 1993 Harwood Academic Publishers GmbH

[B5] Choi MJ Maibach HI Liposomes and niosomes as topical drug delivery systems Skin Pharmacol Physiol 2005 18 209 219 10.1159/000086666 16015019

[B6] Selvamuthukumar S Anandam S kannan K Manavalan R Nanosponges: A novel class of drug delivery system - Review J Pharm Pharmaceut Sci 2012 15 103 111 10.18433/j3k308 22365092

[B7] Smith A Hunneyball lM Evaluation of poly (lactic acid) as a biodegradable drug delivery system for parenteral administration Int J Pharm 1986 30 215 220 10.1016/0378-5173(86)90081-5

[B8] Lherm C Müller RH Puisieux F Couvreur P Alkylcyanoacrylate Drug Carriers II: Cytotoxicity of Cyanoacrylate Nanoparticles with Different Alkyl Chain Length Int J Pharm 1992 84 13 22 10.1016/0378-5173(92)90210-S

[B9] Lucks JS Müller RH Medication vehicles made of solid lipid particles (solid lipid nanospheres SLN), in EP0000605497 1996 Germany

[B10] Muller RH Radtke M Wissing SA Solid lipid nanoparticles (SLN) and nanostructured lipid carriers (NLC) in cosmetic and dermatological preparations Adv Drug Deliv Rev 2002 54 131 155 10.1016/s0169-409x(02)00118-7 12460720

[B11] Saupe A Wissing SA Lenk A Schmidt C Müller RH Solid Lipid Nanoparticles (SLN) and Nanostructured Lipid Carriers (NLC) – Structural investigations on two different carrier systems Bio-Med Mater Eng 2005 15 393 402 16179760

[B12] Muller RH Radtke M Wissing SA Nanostructured lipid matrices for improved microencapsulation of drugs Inte J Pharm 2002 242 121 128 10.1016/S0378-5173(02)00180-1 12176234

[B13] Jenning V Thunemann AF Gohla SH Characterisation of a novel solid lipid nanoparticle carrier system based on binary mixtures of liquid and solid lipids Int J Pharm 2000 199 167 177 10.1016/S0378-5173(00)00378-1 10802410

[B14] Jenning V Mader K Gohla S Solid lipid nanoparticles (SLN) based on binary mixtures of liquid and solid lipids: a 1H-NMR study Int J Pharm 2000 205 15 21 10.1016/S0378-5173(00)00462-2 11000538

[B15] Muller RH Mader K Gohla S Solid lipid nanoparticles (SLN) for controlled drug delivery- a review of the state of the art Eur J Pharm Biopharm 2000 50 161 177 10.1016/S0939-6411(00)00087-4 10840199

[B16] Muller RH Gohla S Dingler A Schneppe T Wise DL Large scale production of solid lipid nanoparticles (SLNTM) and nanosuspensions (DissoCubesTM) Handbook of Pharmaceutical Controlled Release Technology 2000 359 376

[B17] Uner M Preparation, characterization and physico-chemical properties of solid lipid nanoparticles (SLN) and nanostructured lipid carriers (NLC): their benefits as colloidal drug carrier systems Die Pharmazie 2006 61 375 386 16724531

[B18] Bunjes H Westesen K Koch MH Crystallization tendency and polymorphic transition in triglyceride nanoparticles Int J Pharm 1996 129 159 173 10.1016/0378-5173(95)04286-5

[B19] Muller RH Extended patent on the basis of (6), PCT application PCT/EP00/04112 2000

[B20] Muller RH PCT application PCT/EP00/04111 2000

[B21] Radtke M Müller RH Stability study of creams containing cyclosporine SLN™ Int Symp Control Rel Bioact Mater 2001 28 472 473

[B22] Radtke M Müller RH Novel concept of topical cyclosporine delivery with supersaturated SLN™ creams Int Symp Control Rel Bioact Mater 2001 28 470 471

[B23] Freitas C Müller RH Correlation between long-term stability of solid lipid nanoparticles (SLN) and crystallinity of the lipid phase Eur J Pharm Biopharm 1999 47 125 132 10.1016/S0939-6411(98)00074-5 10234536

[B24] Lippacher A Pharmaceutical Characterization of Liquid and Semi-Soid SLN Dispersions for Topical Application PhD Thesis 2001 Germany: Free University of Berlin

[B25] Velmurugan R Selvamuthukumar S Manavalan R Multi criteria decision making to select the suitable method for the preparation of nanoparticles using an analytical hierarchy process Die Pharmazie 2011 66 836 842 22204128

[B26] Ewesuedo RB Ratain MJ Vokes EE, Golomb HM Principles of cancer chemotherapy Oncologic Therapeutics 2003 New York: Springer 19 66

[B27] Early Breast Cancer Trialists' Collaborative Group Polychemotherapy for early breast cancer: an overview of the randomized trials Lancet 1998 352 930 942 9752815

[B28] Ratain MJ Mick R Schilsky RL, Milano GA, Ratain MJ Principles of pharmacokinetics and pharmacodynamics Principles of Antineoplastic Drug Development and Pharmacology 1996 New York: Marcel Dekker 123 142

[B29] Tipton JM Skeel RT Side effects of cancer chemotherapy Handbook of Cancer Chemotherapy 2003 Philedelphia: Lippincott Williams & Wilkins 561 580

[B30] Powis G Powis G, Hacker MP A unique opportunity to study human toxicology The Toxicity of Anticancer Drugs 1991 TorontoL: Pergamon Press 1 9

[B31] Lu P Monitoring cardiac function in patients receiving doxorubicin Nucl Med 2005 35 197 201 10.1053/j.semnuclmed.2005.02.005 16098293

[B32] Kalyanaraman B Joseph J Kalivendi S Wang S Konorev E Kotamraju S Doxorubicin-induced apoptosis: implications in cardiotoxicity Mol Cell Biochem 2002 234 119 124 10.1023/A:1015976430790 12162424

[B33] Hryniuk WA Figueredo A Goodyear M Applications of dose intensity to problems in chemotherapy of breast and colorectal cancer Oncol 1987 11 3 11 3317849

[B34] Gieseler F Rudolph P Kloeppel G Foelsch UR Resistance mechanisms of gastrointestinal cancers: why does conventional chemo- therapy fail? Int J Colorectal Dis 2003 18 470 480 10.1007/s00384-003-0496-x 12774240

[B35] Baird RD Kaye SB Drug resistance reversal - are we getting closer? Eur J Cancer 2003 39 2450 2461 10.1016/S0959-8049(03)00619-1 14602131

[B36] Safa AR Schilsky RL, Milano GA, Ratain MJ Multidrug resistance Principles of Antineoplastic Drug Development and Pharmacology 1996 New York: Marcel Dekker 457 486

[B37] Matsumura Y Maeda H A new concept for macromolecular therapeutics in cancer chemotherapy: mechanism of tumoritropic accumulation of proteins and the antitumor agent Cancer Res 1968 6 193 210 2946403

[B38] Mehnert W Mader K Solid lipid nanoparticles: production, characterization and applications Adv Drug Deliv Rev 2001 47 165 196 10.1016/S0169-409X(01)00105-3 11311991

[B39] Muller RH Mader K Gohla S Solid lipid nanoparticles (SLN) for controlled drug delivery — a review of the state of the art Eur J Pharm Biopharm 2000 50 61 177 10.1016/S0939-6411(00)00075-8 10840199

[B40] Pratt WB Ruddon RW Ensminger WD Maybaum J The Anticancer Drugs 1994 New York: Oxford University Press

[B41] Cavalli R Caputo O Gasco MR Solid lipospheres of doxorubicin and idarubicin Int J Pharm 1993 89 9 12 10.1016/0378-5173(93)90302-V

[B42] Zur Muhlen A Schwarz C Mehnert W Solid lipid nanoparticles (SLN) for controlled drug delivery — drug release and release mechanism Eur J Pharm Biopharm 1998 45 149 155 10.1016/S0939-6411(97)00150-1 9704911

[B43] Cavalli R Caputo O Gasco MR Preparation and characterization of solid lipid nanospheres containing paclitaxel Eur J Pharm Sci 2000 10 305 309 10.1016/S0928-0987(00)00081-6 10838020

[B44] Hahn SM Russo A Cook JA Mitchell JB A multidrug-resistant breast cancer line induced by weekly exposure to doxorubicin Int J Oncol 1999 14 273 279 9917502 10.3892/ijo.14.2.273

[B45] Campone M Vavasseur F Le MT Cabellec K Meflah FM Vallette L Induction of chemoresistance in HL-60 cells concomitantly causes a resistance to apoptosis and the synthesis of P-glycoprotein Leukemia 2001 15 1377 1387 10.1038/sj.leu.2402222 11516098

[B46] Moghimi SM Szebeni J Stealth liposomes and long circulating nanoparticles: critical issues in pharmacokinetics, opsonization and protein-binding properties Prog Lipid Res 2003 42 463 478 10.1016/S0163-7827(03)00033-X 14559067

[B47] Gabizon AA Muggia FM Woodle MC, Storm G Long Circulating Liposomes: Old Drugs, New Therapeutics 1998 Austin, Texas: Springer-Verlag and Landes Bioscience 165 174

[B48] Park JW Liposome-based drug delivery in breast cancer treatment Breast Cancer Res 2002 4 95 99 10.1186/bcr432 12052251 PMC138729

[B49] Zara GP Cavalli R Bargoni A Fundaro A Vighetto D Gasco MR Intravenous administration to rabbits of non-stealth and stealth doxorubicin-loaded solid lipid nanoparticles at increasing concentrations of stealth agent: pharmacokinetics and distribution of doxorubicin in brain and other tissues J Drug Target 2002 10 327 335 10.1080/10611860290031868 12164381

